# In Situ Formation of Compound Eye-like SAN-OSB Composite Microspheres by Melt-Blending Method: Enhancing Multiple-Scattering Effect

**DOI:** 10.3390/polym16142076

**Published:** 2024-07-20

**Authors:** Yuhan Li, Yitong Ding, Yuhao Duan, Fengying Yang, Ying Xiong, Shaoyun Guo

**Affiliations:** 1 State Key Laboratory of Polymer Materials Engineering, Sichuan Provincial Engineering Laboratory of Plastic/Rubber Complex Processing Technology, Polymer Research Institute of Sichuan University, Chengdu 610065, China; 2 Key Laboratory of Biobased Polymer Materials, Shandong Provincial Education Department, College of Polymer Science and Engineering, Qingdao University of Science and Technology, Qingdao 266042, China; dingyitong@qust.edu.cn

**Keywords:** compound eye-like SAN-OSB microspheres, light-diffusing material, melt-blending method, multiple scattering

## Abstract

The preparation of novel structures of light-diffusing particles is currently a research focus in the field of light-diffusing materials. This study, conducted by the common melt-blending process, controlled thermodynamic and kinetic factors to distribute smaller-sized organic silica bead (OSB) particles at the interface between a polycarbonate (PC) matrix and spherical island-phase styrene–acrylonitrile copolymer (SAN) for the in situ formation of compound eye-like microspheres with SAN as “large eyes” and OSBs as “small eyes”. Through the multiple-scattering effects of these compound eye-like microspheres, these light-diffusing materials significantly improved the haze, scattering range, and light-shielding capabilities while maintaining high transmittance. Specifically, the PC/SAN-OSB light-scattering materials achieved a haze of 100% with an OSB content of only 0.17%, maintaining a transmittance of 88%. Compared with the PC/OSB system with the same level of haze, the addition of OSB was reduced by 88%. Therefore, this study achieved exceptionally effective light-diffusing materials through a simple, environmentally friendly, and low-cost preparation method, suitable for the scalable production of light-diffusing materials in new display and lighting fields.

## 1. Introduction

The rapid advancement and diverse applications of large-screen displays and mini- light emitting diode (LED) have intensified the demand for polymer-based light-scattering materials, which are indispensable in LED lighting and display systems. These materials are not only expected to deliver superior transmittance and haze but also offer an extensive effective scattering range along with uniform scattering characteristics [1,2]. Incorporating light diffusing particles (LDPs) represents a practical approach to enhancing the light-scattering performance of materials. Organic particles, such as acrylic and organic silica bead (OSB) microspheres [3], are the most common LDPs and offer controllable optical properties and better compatibility with polymers. OSB microspheres, distinguished by superior mechanical properties and thermal stability, enjoy widespread market applications [4]. However, due to the haze and light-shielding capabilities required for applications, the addition of OSB microspheres needs to be controlled within the range of 1% to 5%. But at this addition level, the transmittance during application is significantly reduced to nearly 58% [5]. Adjusting the structure of scattering particles to induce multiple-scattering effects may alter this condition.

The intricate structure of an insect’s compound eye, known for its exceptional optical sensitivity and imaging resolution [6,7], serves as a natural blueprint for optimizing the light properties of materials. Peng et al. [8] leveraged UV nanoimprint lithography to create multiscale compound eye arrays on polyethylene terephthalate (PET) substrates, achieving a remarkable 90% transmittance across the 400–800 nm spectrum. Similarly, Wang et al. [9] developed a hierarchical compound eye array using an integrated manufacturing approach, furthering the practical application of these biomimetic structures. These studies showed that compound eye-like structures minimize surface reflections and boost photosensitivity [8,10], while their orderly microscale arrangement extends the effective light path, enhancing diffuse light transmission without compromising total transmittance [11,12,13,14]. However, these constructions of complex scattering particles often involve solvent use, complex manufacturing processes, and expensive equipment, often employing coating methods for the integration with the substrate [15,16,17]. This limits the fabrication and subsequent application scenarios of light-scattering materials.

Melt blending, a simple, environmentally friendly, and low-cost processing method, is commonly used for preparing light-scattering materials. In melt blending, thermodynamic theory can preliminarily predict the migration distribution of fillers in polymer blends, and the controlled orientation distribution of fillers to construct micro/nanostructures can be achieved by adjusting kinetic parameters [18,19,20]. Ding et al. [21] achieved the phase distribution of SiO_2_ within styrene–acrylonitrile copolymer (SAN) by selecting material systems with appropriate wettability coefficients, resulting in a light-scattering material with pomegranate-like scattering microspheres that enhanced the effective scattering range and uniformity of the polycarbonate (PC) matrix. This opens up new ideas for the simple preparation of scatterers with compound eye-like structures and their application in light-scattering materials.

Previous research by our team found that SAN with an acrylonitrile (AN) content of 20–25%wt. could distribute as spherical island-phase structures within a PC matrix, with a particle size of 2–10 μm, ideal for serving as “large eyes” in compound eye-like microspheres [22,23]. Building on this foundation, OSB particles, with a small diameter and appropriate surface wettability, were synthesized in this study. By controlling thermodynamic principles and kinetic factors, these smaller OSB particles were distributed at the interface between a PC matrix and island-phase SAN for the in situ fabrication of compound eye-like composite microspheres with SAN as “large eyes” and OSB particles as “small eyes” during the melt-blending process. These composite microspheres induce multiple scattering, reducing direct light transmission and enhancing scattering transmittance. This results in increased haze and a broader scattering angle without compromising the material’s overall light transmittance, effectively improving the scattering uniformity and the effective scattering range. This fabrication method is simple, economically efficient, and environmentally friendly, with low addition amounts of OSBs, significantly reducing the cost of producing light-diffusing materials and facilitating their large-scale application.

## 2. Materials and Methods

### 2.1. Materials and Preparation

**Figure 1:**
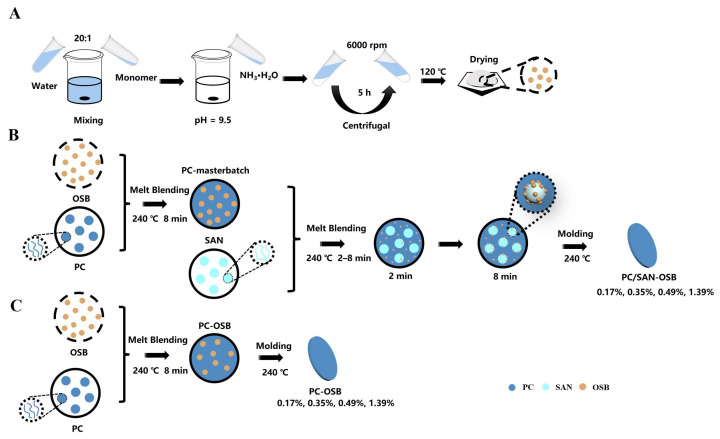
(**A**) Schematic diagram of OSB preparation process, (**B**) schematic diagram of PC/SAN-OSB composite preparation process, and (**C**) schematic diagram of PC/OSB composite preparation process.

PC and SAN were purchased from LG DOW Polycarbonate Ltd. (Yosu, Republic of Korea) and Formosa Plastics (Ningbo) Ltd. (Ningbo, China), respectively. OSB microspheres were synthesized via a one-step solution-based approach [4], as delineated in Figure 1A. A 20:1 mixture of deionized water–methyltrimethoxysilane was stirred until homogeneous. The pH was adjusted to 9.5 with 25% ammonium hydroxide and stirred at 25 °C for 5 h. The resultant product was then centrifuged, washed, and vacuum-dried to obtain the OSB microspheres.

To ensure moisture-free conditions, the PC, SAN, and OSBs were vacuum-dried at 80 °C for 12 h. The PC/SAN-OSB light-scattering sheets’ fabrication involved melt mixing the dried materials using a torque rheometer (RM-200C, Harbin Harper Electric Technology Co., Ltd., Harbin, China) and subsequently processing them with a molding machine (R3212, Wuhan Qion Science and Technology Development Co., Ltd., Wuhan, China), as illustrated in Figure 1B. The PC/OSB composites were created by melt blending PC and OSBs at various ratios. These blends were then combined with SAN at a 7:3 ratio, undergoing blending durations of 2 min, 4 min, and 8 min at 240 °C. The blends were molded into sheets of a 0.6 mm thickness at 240 °C, designated as x–y, where x-values of 0 and 1 represented PC/OSB and PC/SAN-OSB, respectively. The y-values (1, 2, 3, or 4) corresponded to the OSB contents (g cm^−3^), indicating OSB percentages of 0.17%, 0.35%, 0.49%, and 1.39%, as per Equation (1):

(1) $$n_{0}=\frac{m}{V_{A}}\times 100\%$$

where in m and $V_{A}$ are the added mass of OSBs and the total added volume (48 mL) of the material, respectively.

### 2.2. Characterization Technique

The contact angles (water and diiodomethane) were measured using a DSA 25S optical contact angle goniometer at 25 ± 2 °C, with each sample measured 5 times. The cross-sectional morphologies of the PC/SAN-OSB and PC/OSB composites were examined using a scanning electron microscope (SEM, JSM5900LV, Japan Electronics Co., Ltd., Tokyo, Japan), with the version 1.2 Nano-measurer software [24] employed for particle size analysis of the SAN phase and OSB particles.

The transmittance (T) and haze (H) of the samples were measured using a WGT-S Transmittance/Haze Meter (Shanghai Precision Scientific Instrument Co., Ltd., Shanghai, China) acquired from, according to the GB/T 2410-2008 standard [25].

Transmitted light (I_T_) is categorized into direct transmission (I_d_) and scattered transmission (I_S_). The direct and scattered transmittances were measured using a UV–Vis spectrophotometer (UV-3600, Shimadzu (China) Co., Ltd., Shanghai, China) equipped with an integrating sphere, with the formulas for the total transmittance (T_T_), direct transmittance (Td), and scattered transmittance (T_S_) presented in Equations (2) and (3), over a wavelength range of 400–800 nm.

(2) $$I_{T}=T_{S}+T_{d}=I_{S}/I_{0}+I_{d}/I_{0}$$

(3) $$T_{T}=T_{S}+T_{d}=I_{S}/I_{0}+I_{d}/I_{0}$$

**Figure 2:**
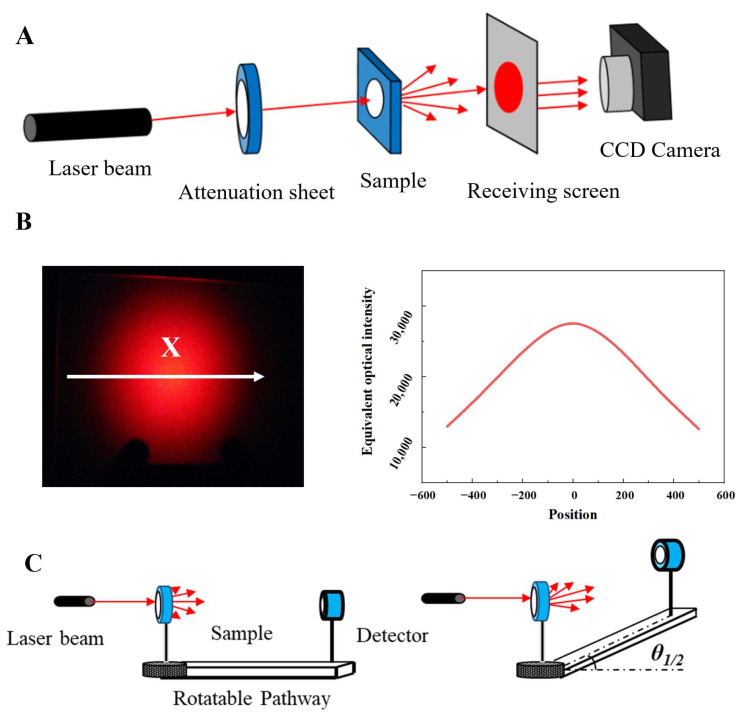
(**A**) Schematic diagram of diffusing pattern measuring setup, (**B**) analytical method for equivalent optical intensity, and (**C**) schematic diagram of scattering angle testing setup and seeking out the half angle [21].

The scattering pattern was measured using a custom-built platform, as depicted in Figure 2A, with a 632.8 nm He–Ne laser as the light source. The light, attenuated and passed through the sample, was received by a screen and recorded by a CCD camera. A schematic of the scattering pattern is shown in Figure 2A. The scattering pattern was processed with the Origin 9.0 software [26] to generate a scattering curve by converting the intensity of each point in the pattern into pixel numbers, with the curve comprising the distance of each point from the center of the scattering pattern and the intensity of the point [21]. The specific values were obtained by converting the scattering spots into grayscale images and then using the contour function in Origin for the calculation.

The scattering angle is defined as the angular difference at which the light intensity is half of the peak value, which was measured using a custom-built platform, as shown in Figure 2B, with the same 632.8 nm He–Ne laser as the light source and an SZX-type light power meter placed 485 nm from the sample on the opposite side. Due to the symmetrical Gaussian distribution of scattered light, the scattering angle of the sample can be represented as 2$\theta_{1/2}$, as shown in Equation 4. The procedure involved aligning the laser source with the center of the light power meter receiver at the same height, then rotating the platform to record the angle $\theta_{0}$ corresponding to the maximum power meter reading, and, again, rotating the platform to record the angle θ when the power reached half of its maximum value.

(4) $${\;\theta }_{1/2}=\left|\theta -\theta_{0}\right|$$

Mechanical properties, including tensile and impact strength, were evaluated using a universal material testing machine (Instron 5567, Illinois Tool Works Inc., Norwood, MA, USA) and an impact testing machine (XJU-22, Shanghai Jiezhun Instrument Equipment Co., Ltd., Shanghai, China), respectively, at a controlled temperature of 25 ± 2 °C, following the GB/T1040-1992 and GB/T1043.1-2008 standards [27,28].

## 3. Results

### 3.1. The Preparation of “Small Eyes” (OSB Microspheres) and the Construction of “Compound Eye-like” Composite Microspheres

**Figure 3:**
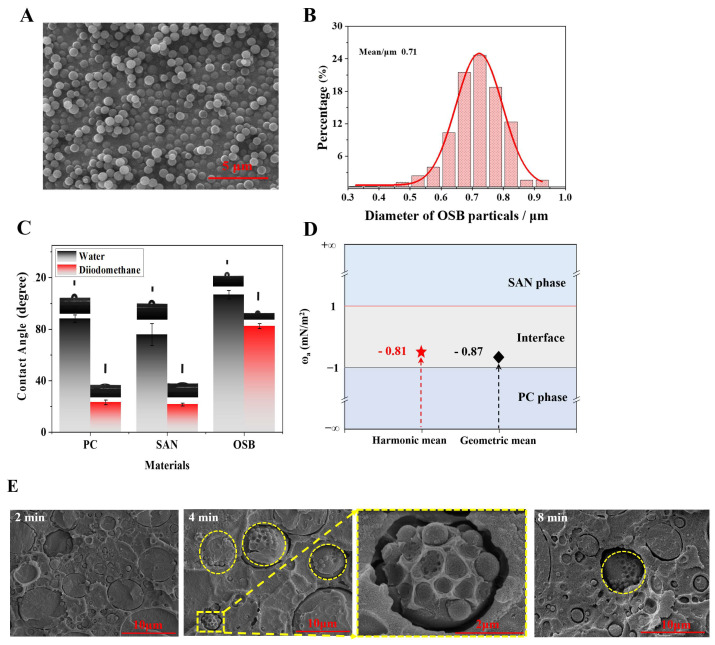
(**A**) SEM micrographs of OSB samples, (**B**) average particle size and size distribution of OSB particles, (**C**) contact angles of PC, SAN, and OSB at the water and diiodomethane interfaces, (**D**) thermodynamic selective decentralized prediction of OSB particles in the PC/SAN mixture, and (**E**) SEM of PC/SAN-OSB samples after 2 min, 4 min, and 8 min of melt blending.

Our objective was to synthesize OSB organic spheres, dubbed “small eyes”, to enhance the light-scattering capabilities of the material by emulating the compound eye structure of insects. The SEM-characterized structure of the OSBs revealed smooth, spherical surfaces (Figure 3A). The particle size distribution was mostly concentrated in the range of 0.6–0.8 μm, with an average particle size of 0.71 µm, as seen in Figure 3B. The smaller particle size of OSBs compared with those of SAN spheres [22] (2–10 µm) allows for an ample dispersion space for the OSB spheres. Additionally, the refractive index of OSBs (1.385) is suitably lower than that of SAN (1.567) and PC (1.586), making OSBs an appropriate secondary scatterer due to the moderate differences in the refractive indices.

Thermodynamic theory was employed to assess the compatibility of PC, SAN, and OSBs. Fillers in polymer blends tend to selectively disperse in components with which they have stronger interactions, assessable by the wettability coefficient *ωa*. The contact angle results of PC, SAN, and OSBs in water and methylene iodide are presented in Figure 3C and can be calculated and compared according to Equation (5) [29]:

(5) $$\omega_{a}=\frac{\gamma_{\mathrm{filler}/\mathrm{polymer}1}-\gamma_{\mathrm{filler}/\mathrm{polymer}2}}{\gamma_{\mathrm{polymer}1/\mathrm{polymer}2}}$$

wherein $\gamma_{\mathrm{filler}/\mathrm{polymer}1}$, $\gamma_{\mathrm{filler}/\mathrm{polymer}2}$, and $\gamma_{\mathrm{polymer}1/\mathrm{polymer}2}$ are the interfacial energies of the OSBs and PC, OSBs and SAN, and PC and SAN, respectively. The calculations of the interfacial tension followed Equation (6) (the harmonic mean equation) and Equation (7) (the geometric mean equation) [29]:

(6) $$\gamma_{12}=\gamma_{1}+\gamma_{2}-2\;\left(\sqrt{\gamma_{1}^{d}\gamma_{2}^{d}}+\sqrt{\gamma_{1}^{p}\gamma_{2}^{p}}\right)$$

(7) $$\gamma_{12}=\gamma_{1}+\gamma_{2}-4\;\left(\frac{\gamma_{1}^{d}\gamma_{2}^{d}}{\gamma_{1}^{d}+\gamma_{2}^{d}}-\frac{\gamma_{1}^{p}\gamma_{2}^{p}}{\gamma_{1}^{p}+\gamma_{2}^{p}}\right)$$

where in $\gamma_{12}$ represents the interfacial tension between the two components; $\gamma_{1}$ and $\gamma_{2}\;$represent the surface energies of polymer 1 (PC) and polymer 2 (SAN), respectively; $\gamma_{1}^{d}\;$and${\;\gamma }_{2}^{d}$ represent the dispersive parts of the surface energies; and $\gamma_{1}^{p}$ and $\gamma_{2}^{p}\;$represent the polar parts of the surface energies. The values of the surface tension for PC, SAN, and OSB were calculated and are shown in Table 1.

When $-1<\omega_{a}<1$, OSBs were selectively dispersed at the interface between the PC and SAN phases. The results of the $\omega_{a}$ calculations for the PC/SAN-OSB compounds are represented in Figure 3D, predicting that OSBs were more likely to be selectively distributed at the interface between the PC and SAN phases. This demonstrates the feasibility of using PC-SAN/OSB to construct a compound eye structure.

Previous research by our team confirmed that when fabricating PC/SAN composites via melt blending, SAN exists within the PC matrix as spherical sea island structures, particularly at a SAN content of 30%, achieving the optimal performance (Figure S1) [22,23,30]. Building on this, this study further introduced custom-made OSB particles with wettability coefficients ranging from −1 to 1. The aim was to distribute these OSB particles at the interface between the PC and SAN during the melt-blending process, thereby forming in situ compound eye-like microspheres, with SAN as the “large eyes” and OSBs as the “small eyes”. Figure 3E shows the microstructures of the brittle fracture surfaces of the PC/SAN-OSB samples at an OSB content of 1.39% at different melting times (2 min, 4 min, and 8 min). The findings indicate that at 2 min of melt blending, the OSBs were predominantly distributed within the PC matrix. After extending the blending time to 4 min, the OSB particles migrated to the interface between the PC and SAN, forming composite particles with a compound eye-like structure. As the mixing time was further extended to 10 min (Figure S2), the OSB particles remained at the interface instead of migrating to the SAN phase, in agreement with the thermodynamic predictions.

### 3.2. The Impact of Compound Eye-like San-Osb Composite Microspheres on PC Performance

**Figure 4:**
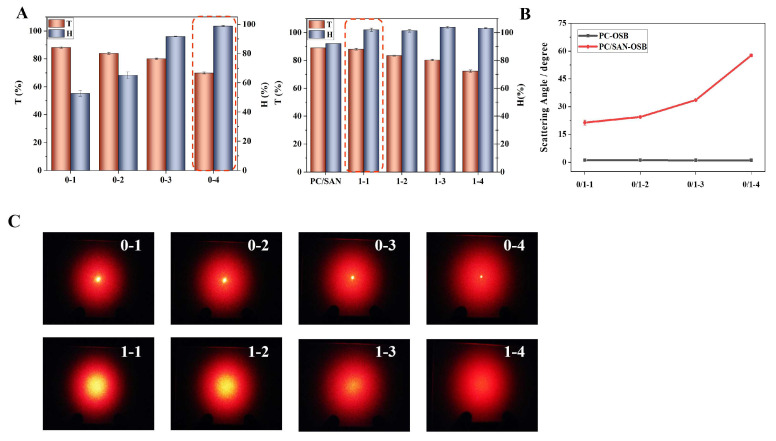

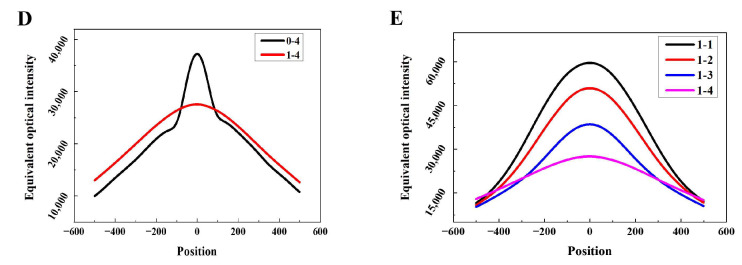
(**A**) Transmittance and haze, (**B**) scattering angles, (**C**) scattering patterns, (**D**,**E**) scattering profiles of PC/OSB (0-1 and 0-4) and PC/SAN-OSB (1-1 to 1-4) composite materials.

The PC/SAN-OSB samples with compound eye-like structures had significantly improved transmittance, haze, and scattering uniformity and an effective scattering range compared with the PC/OSB samples. Investigations into the effect of the “small eye” OSB content on the light diffusion performance of PC/SAN-OSB were conducted (Figure 4). As Figure 4A shows, with an OSB content of merely 0.17%, the haze reached 100%, at which point the transmittance of the sample was as high as 88%, far exceeding that of the PC/OSB system with the same OSB content (55%), representing a 60% improvement. To achieve the same level of haze, the PC/OSB system required an OSB addition of 1.39%, over eight times that of the PC/SAN-OSB system containing compound eye-like microspheres, and its transmittance decreased to 69%. Compared with the PC/SAN without OSBs, the inclusion of OSBs increased the haze from 92% to 100% while maintaining the level of light transmittance. The PC/SAN-OSB system with a compound eye-like structure significantly enhanced the scattering angle (Figure 4B) and markedly intensified the diffusion effect on the point light sources (Figure 4C). Even when the OSB content reached 1.39%, resulting in a haze of 100% in the PC/OSB system, it still failed to shield the light sources, with the light-scattering curve showing a distinct convex profile (Figure 4D). In contrast, the PC/SAN-OSB system forming a compound eye-like structure, even with only a 0.17% OSB addition, effectively scattered the point light, producing a smooth light-scattering curve with a clear Gaussian distribution. As the amount of the “small eye” OSB particles increased, the central brightness gradually decreased, leading to more uniform scattering brightness (Figure 4E). This was attributed to the stronger multiple scattering generated by the compound eye-like microspheres in the PC/SAN-OSB, significantly enhancing its shielding and anti-glare capabilities. Compared with literature-reported scattering particles, the compound eye-like PC/SAN-OSB composite exhibited excellent haze and transmittance at a low addition level [17,31,32,33,34,35].

**Figure 5:**
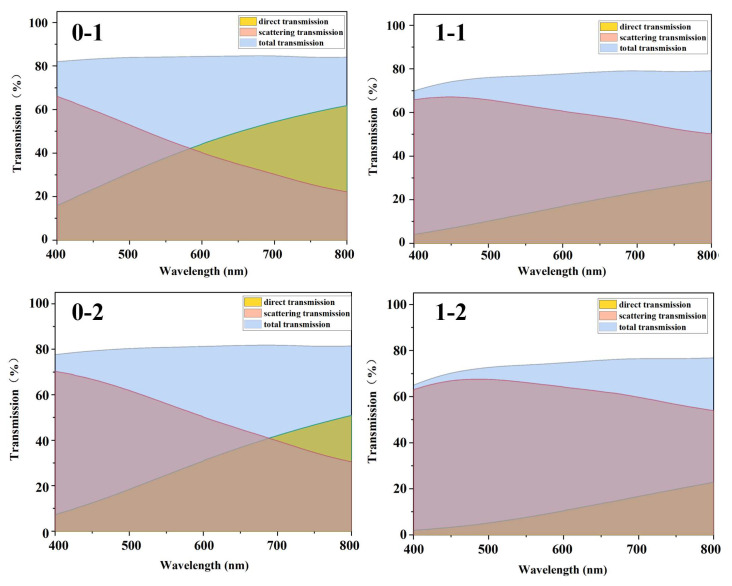

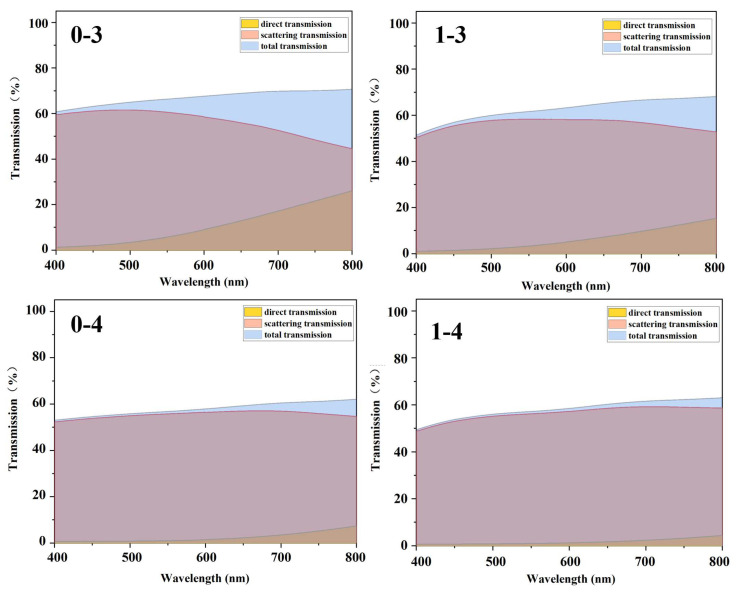
Direct transmittance, scattered transmittance, and total transmittance of PC/OSB (0-1 to 0-4) and PC/SAN-OSB (1-1 to 1-4) composite materials.

Additionally, as observed in the transmittance variation–wavelength graphs (Figure 5), the transmittance of the PC/SAN-OSB system with compound eye-like microspheres was primarily contributed by the scattering transmittance, which increased with the OSB content. In contrast, for the PC/OSB system with the same OSB content, the transmittance was mainly due to direct transmittance. Although the contribution of direct transmittance decreased with an increasing OSB content, its proportion remained higher than that of the PC/SAN-OSB system containing compound eye-like microspheres. This can be attributed to the stronger multiple scattering induced by the compound eye-like microspheres in the PC/SAN-OSB system, reducing the backward scattering of the scattering particles. This enhancement in the scattering transmission, while maintaining high light diffusion, benefits the PC/SAN-OSB system. The increase in the scattering transmittance aids in improving the haze, scattering angle, and anti-glare capabilities of the system, as well as in shielding light sources, which is highly advantageous for the application of light-diffusing materials. Moreover, the compound eye-like microspheres involved in this product are formed in situ during the melt-blending process of transparent PC. The preparation process is simple and environmentally friendly. The low addition amount of OSBs significantly reduces the production cost of light-diffusing materials, facilitating their widespread application.

**Figure 6:**
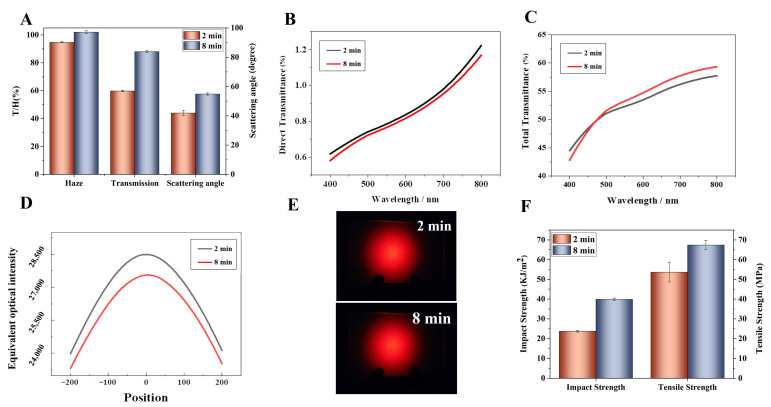
The impact of OSB distribution location on the performance of PC/SAN-OSB composite materials (2 min, within the PC phase; 8 min, at the interface between PC and SAN, forming a compound eye-like structure). (**A**) Comparison of optical performance, (**B**) direct transmittance, (**C**) total transmittance, (**D**) scattering profiles, (**E**) scattering patterns, and (**F**) mechanical performance.

The superior optical performance of PC/SAN-OSB is not solely attributable to the refractive index matching of the system materials. A crucial factor is the formation of a compound eye-like structure. The extent of this structure’s formation was significantly influenced by the blending time. The optical properties of the samples with different blending times are shown in Figure S3. As the melt-blending time increased, the scattering angle of the samples gradually increased and tended to be flat at 8 min, and the transmittance and haze showed similar trends. Combined with the above SEM results, we selected the 2 min and 8 min samples to illustrate the effect of compound eye-like structures on the optical properties of the samples. Compared with the 2 min samples, where the OSBs were distributed within the PC matrix, the 8 min samples, where the OSB particles were distributed at the PC and SAN interface, forming compound eye-like structures, exhibited a 4.72% increase in light transmittance, achieved 100% haze (as shown in Figure 6A), and saw a 57.63° increase in the scattering angle, representing a 28.24% improvement in the scattering angle. Concurrently, for the PC/SAN-OSB samples forming compound eye-like structures, a reduction in the direct light transmittance within the visible spectrum was observed (as indicated in Figure 6B), alongside an enhancement in the total transmittance (Figure 6C), a substantial broadening of the scattering range, and an improvement in the scattering uniformity, evidenced by the diminished brightness at the center of the scattering spot in the 8 min samples (Figure 6D). To further clarify the distinction between the two, the scattering spots were analyzed using the Origin 9.0 software, and the central region was magnified (as shown in Figure 6E). This analysis revealed a noticeable reduction in the mid-section bulge of the scattering curve for the 8 min samples, indicating an enhanced shielding performance with the refinement of the compound eye-like structure. This improvement can be attributed to the stronger multi-scattering effect produced by the compound eye-like microspheres within PC/SAN-OSB while maintaining high light diffusion, which increased its scattering transmission.

Meanwhile, when OSBs were distributed at the interface between the PC and SAN, the number of defects in the PC phase decreased, leading to improvements in the material’s mechanical properties. Compared with the 2 min samples, the 8 min samples exhibited a 67.83% increase in impact strength (39.91 KJ/m^2^) and a 25.64% increase in tensile strength (67.37 MPa), as illustrated in Figure 6F. Above all, the formation of compound eye-like structures not only effectively improved the light-scattering performance of the PC/SAN-OSB system but also increased its mechanical properties.

### 3.3. Light-Diffusing Enhancement Mechanism of Compound Eye-like Composite Microspheres

M. Kerker noted that scatterer distances of less than three times their average diameter are necessary for multiple scattering [36,37]. When multiple scattering occurs, the secondary electrons generated by the light excitation of the scatterers interact, involving more scattering particles in the process and leading to enhanced scattering effects. To understand the influence of compound eye-like structures on light-scattering performance, we analyzed and compared the scattering mechanisms of different structures.

**Figure 7:**
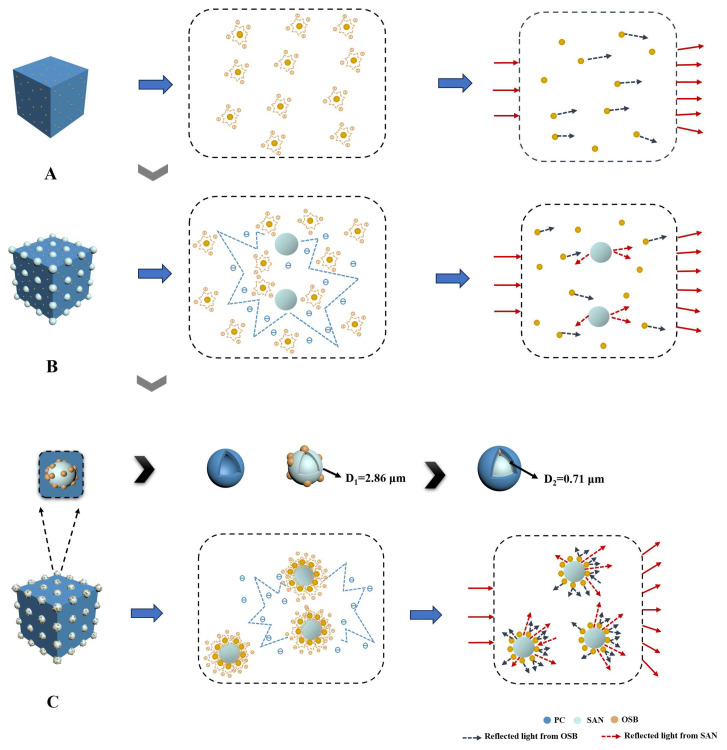
Schematic diagram of the morphology and the scattering mechanism of the samples. (**A**) PC/OSB, (**B**) PC/SAN/OSB (OSBs distributed within the PC matrix), (**C**) PC/SAN-OSB (OSBs distributed at the interface between PC and SAN, forming a compound eye-like structure).

Figure 7A–C display the microstructures and scattering mechanisms for the three different samples, PC/OSB, PC/SAN/OSB (with OSBs distributed within the PC matrix), and PC/SAN-OSB (with OSBs distributed at the interface between the PC and SAN, forming a compound eye-like structure), respectively. The dispersion state of OSB microspheres greatly affects the improvement of the multiple-scattering effect. In the PC/OSB sample, the OSB particles dispersed in the PC phase were distant, requiring a higher content for multiple-scattering effects. Previous experiments demonstrated that when the mass ratio of PC–SAN is 7:3, PC/SAN composites can exhibit an “island” structure [22,23]. The SAN phase meets the conditions for multiple-scattering effects, with the addition of OSBs further promoting these effects. The multiple scattering in the PC/SAN/OSB sample where OSBs were distributed within the PC matrix resulted from interactions between secondary electrons of the OSB particles in the PC phase and between the OSBs and SAN particles. In the PC/SAN-OSB sample where OSBs were distributed at the interface between the PC and SAN, two sources of multiple scattering existed: interactions between secondary electrons generated by the OSB particles dispersed at the PC and SAN interface, and interactions between secondary electrons generated at the interface and by the OSB particles. To quantitatively compare the degree of multiple-scattering effects between different samples, we introduced the concept of confined volume (V_A_, V_B_, and V_C_)^3^, calculated as shown in Equations (8)–(10). The confined volume ratios of the three samples can be expressed as V_A_:V_B_:V_C_ = 48:32:24 = 6:4:3. A lower confined volume leads to reduced particle separation, converting more backward scattering into forward scattering, thereby providing a stronger multiple-scattering effect.

(8) $$N=\frac{V_{0}}{V_{1}}$$

(9) $$V_{\mathrm{SAN}}=V_{T}-V_{\mathrm{PC}}$$

(10) $$V_{\mathrm{Con}}=\frac{4}{3}\pi N\left[{\left(\frac{D_{1}+D_{2}}{2}\right)}^{3}-{\left(\frac{D_{1}-D_{2}}{2}\right)}^{3}\right]$$

where in N is the theoretical number of SAN microspheres. $V_{T}\;$is the total added volume of the primary material (48 mL). $V_{\mathrm{PC}}$ and $V_{\mathrm{SAN}}$ are the volume of PC and the volume of SAN (32 mL and 16 mL), respectively. $D_{2}$ is the average diameter of a single OSB particle (0.71 μm). $V_{\mathrm{Con}}$ is the restricted volume when OSB particles are selectively distributed at the interface between PC and SAN. $V_{1}$ and $D_{1}\;$are the average volume and average diameter (2.86 μm) of a single SAN microsphere, respectively.

## 4. Discussion

In this study, during the melt-blending process of PC/SAN, OSB particles with wettability coefficients ranging from −1 to 1 were introduced and strategically positioned at the interface between the PC matrix and the dispersed SAN microspheres, forming compound eye-like microspheres with SAN as the “large eyes” and OSB particles as the “small eyes”. These composite microspheres significantly enhanced the light diffusion through their multiple-scattering effects. When the OSB content was only 0.17%, the sample maintained a transmittance of 88% and achieved a haze of 100%, representing a 60% improvement in the haze compared with the PC/OSB samples with the same OSB content. Additionally, compared with the PC/OSB system at the same level of haze, the addition of OSBs was reduced by 88%. Moreover, in the PC/SAN-OSB samples containing compound eye-like microspheres, the transmittance was predominantly contributed by the scattering transmittance, which is highly beneficial for reducing glare and shielding light sources. Overall, this study presents a simple, environmentally friendly, and low-cost method for producing light-scattering materials, significantly reducing production costs and facilitating their large-scale application. Given these attributes, the developed materials hold promising potential for applications in mini-LEDs and large-screen display technologies.

## Figures and Tables

**Table 1:** Thermodynamic selective decentralized prediction of OSB particles in the PC/SAN mixture.

Materials	$\gamma_{1}$	$\gamma_{2}$	$\gamma_{12}—$Harmonic	$\gamma_{12}$—Geometric
PC/OSB	47.36	17.21	7.93	14.86
SAN/OSB	50.72	17.21	8.85	16.55
PC/SAN	47.36	50.72	1.13	1.94

## Data Availability

The original contributions presented in this study are included in this article/the Supplementary Materials. Further inquiries can be directed to the corresponding authors.

## Supplementary Materials

The following supporting information can be downloaded at https://www.mdpi.com/article/10.3390/polym16142076/s1. Figure S1: Effect of the content of SAN on the transmittance and haze; Figure S2: SEM of samples at different processing times. (A–E) represent 2 min, 4 min, 6 min, 8 min, and 10 min, respectively; Figure S3: (A) Transmittance and haze of samples at different processing times. (B) Scattering angles of samples at different processing times.
