# Supplementary material for: In Situ Formation of Compound Eye-like SAN-OSB Composite Microspheres by Melt-Blending Method: Enhancing Multiple-Scattering Effect
Source: Polymers (Basel). 2024 Jul 20;16(14):2076. doi: 10.3390/polym16142076 (PMC11281021; doi:10.3390/polym16142076)
Supplement: Supplementary file 1 [file polymers-16-02076-s001.zip › polymers-3095159-supplementary.pdf]

Supporting Information

In Situ Formation of Compound Eye-like SAN-OSB Composite Microspheres by Melt-Blending Method: Enhancing Multiple-Scattering Effect

Yuhan Li [1], Yitong Ding [2], Yuhao Duan [1], Fengying Yang [1], Ying Xiong [1,*] & Shaoyun Guo [1,*]

[1] State Key Laboratory of Polymer Materials Engineering, Sichuan Provincial Engineering Laboratory of Plastic/Rubber Complex Processing Technology, Polymer Research Institute of Sichuan University, Chengdu 610065, China

[2] Key Laboratory of Biobased Polymer Materials, Shandong Provincial Education Department, College of Polymer Science and Engineering, Qingdao University of Science and Technology, Qingdao 266042, China

* Corresponding author. Tel/Fax: 86-028-85466255, Email: xiongying@scu.edu.cn (Ying Xiong).

* Corresponding author. Tel/Fax: 86-028-85466255, Email: nic7702@scu.edu.cn (Shaoyun Guo).

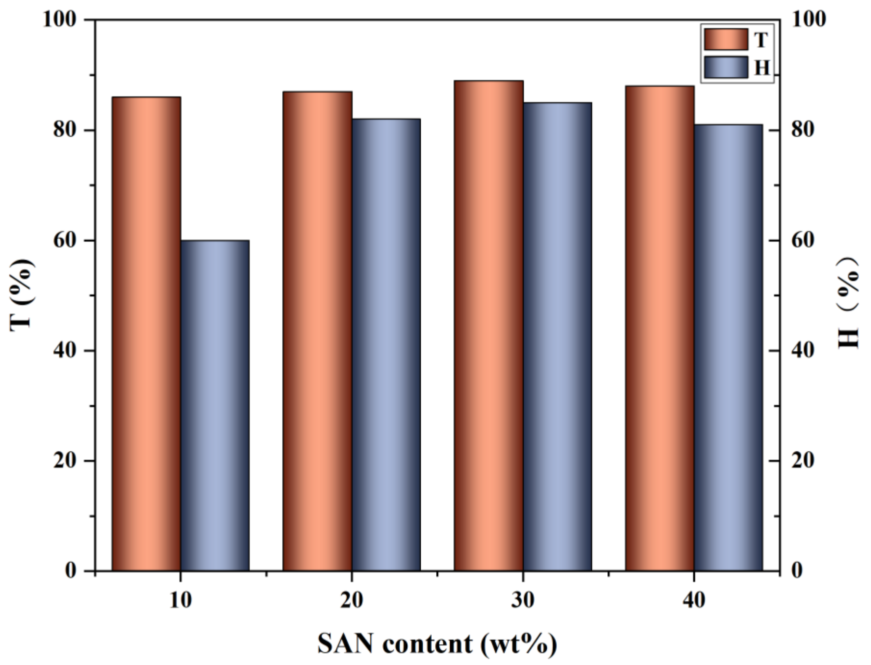

Figure S1. Effect of the content of SAN on the transmittance and haze.

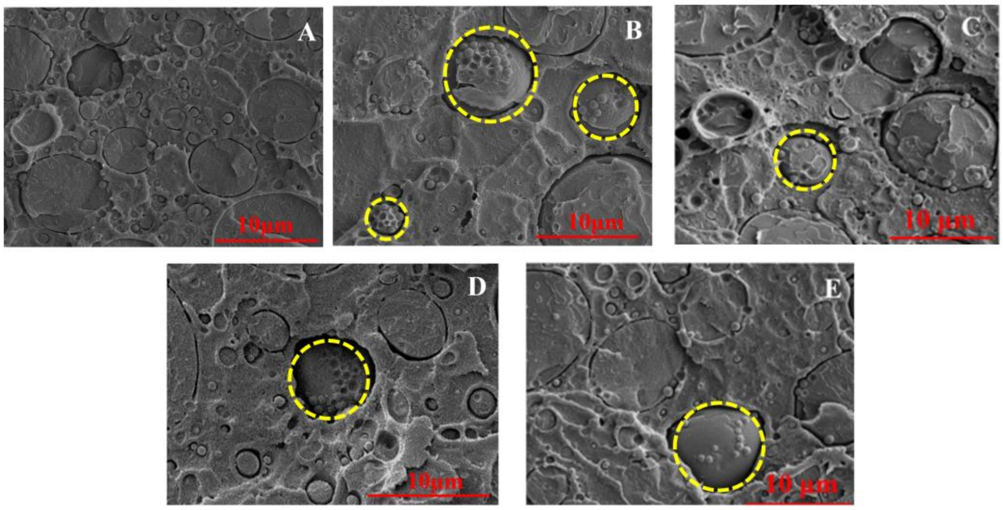

Figure S2. SEM of samples at different processing times. (A-E) represent 2min, 4min, 6min,8min and 10min respectively.

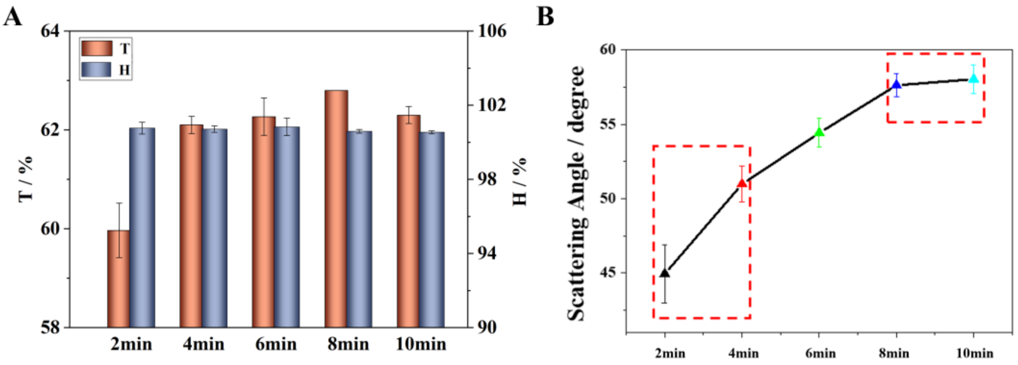

Figure S3. (A) Transmittance and haze of samples at different processing times. (B) Scattering angles of samples at different processing times.
